# Antifungal and Antibiofilm Efficacy of Paeonol Treatment Against Biofilms Comprising *Candida albicans* and/or *Cryptococcus neoformans*

**DOI:** 10.3389/fcimb.2022.884793

**Published:** 2022-05-20

**Authors:** Weidong Qian, Xinchen Li, Qiming Liu, Jiaxing Lu, Ting Wang, Qian Zhang

**Affiliations:** ^1^ School of Food and Biological Engineering, Shaanxi University of Science and Technology, Xi’an, China; ^2^ Department of Dermatology, Huazhong University of Science and Technology Union Shenzhen Hospital, Shenzhen, China

**Keywords:** *Candida albicans*, paeonol, antibiofilm, *Cryptococcus neoformans*, RNA sequencing

## Abstract

Fungal populations are commonly found in natural environments and present enormous health care challenges, due to increased resistance to antifungal agents. Paeonol exhibits antifungal activities; nevertheless, the antifungal and antibiofilm activities of paeonol against *Candida albicans* and *Cryptococcus neoformans* remain largely unexplored. Here, we aimed to evaluate the antifungal and antibiofilm activities of paeonol against *C. albicans* and/or *C. neoformans* (i.e., against mono- or dual-species). The minimum inhibitory concentrations (MICs) of paeonol for mono-species comprising *C. albicans* or *C. neoformans* were 250 μg ml^−1^, whereas the MIC values of paeonol for dual-species were 500 μg ml^−1^. Paeonol disrupted cell membrane integrity and increased the influx of gatifloxacin into cells of mono- and dual-species cells, indicating an antifungal mode of action. Moreover, paeonol at 8 times the MIC damaged mono- and dual-species cells within *C. albicans* and *C. neoformans* biofilms, as it did planktonic cells. In particular, at 4 and 8 mg ml^−1^, paeonol efficiently dispersed preformed 48-h biofilms formed by mono- and dual-species cells, respectively. Paeonol inhibited effectively the yeast-to-hyphal-form transition of *C. albicans* and impaired capsule and melanin production of *C. neoformans*. The addition of 10 MIC paeonol to the medium did not shorten the lifespan of *C. elegans*, and 2 MIC paeonol could effectively protect the growth of *C. albicans* and *C. neoformans*-infected *C. elegans*. Furthermore, RNA sequencing was employed to examine the transcript profiling of *C. albicans* and *C. neoformans* biofilm cells in response to 1/2 MIC paeonol. RNA sequencing data revealed that paeonol treatment impaired biofilm formation of *C. albicans* by presumably downregulating the expression level of initial filamentation, adhesion, and growth-related genes, as well as biofilm biosynthesis genes, whereas paeonol inhibited biofilm formation of *C. neoformans* by presumably upregulating the expression level of ergosterol biosynthesis-related genes. Together, the findings of this study indicate that paeonol can be explored as a candidate antifungal agent for combating serious single and mixed infections caused by *C. albicans* and *C. neoformans*.

## Introduction

Outbreaks of fungal infections have still plagued healthcare settings over recent years, especially among hospitalized patients with weakened immune systems and those who are immunocompromised (Delaloye and Calandra, 2014; Van Schalkwyk et al., 2018; Rodrigues and Nosanchuk, 2020). The causative pathogens for these infections are usually *Candida* and *Cryptococcus* species (Netea et al., 2015; Iyer et al., 2021). The genus *Candida* embraces around 154 species. Among these, *Candida albicans* (*C. albicans*) is the most common cause of hospital-acquired, invasive fungal infections such as candidemia, and the mortality rate of candidemia approaches 40% (Fux et al., 2005; Tampakakis et al., 2009). *C. albicans* can cause mainly two types of infections, ranging from superficial infections, including oral or vaginal candidiasis, to life-threatening systemic infections (Mayer et al., 2013; Allert et al., 2018). Previous studies demonstrate that the pathogenic potential of this fungus has mainly been associated with several well-established virulence factors, including biofilm formation, the secretion of virulence proteins, and motility factors, such as swimming and swarming (Gulati and Nobile, 2016; Abirami et al., 2020). Furthermore, a growing body of evidence shows that the formation of biofilm and appendix has been implicated in pathogenicity as they can act as reservoirs for persister cells of *C. albicans*, thus driving recurrent candidemia (Lafleur et al., 2006; Wu et al., 2020).

The genus *Cryptococcus* includes over 30 species, but two species, in particular, *Cryptococcus neoformans* (*C. neoformans*) and *Cryptococcus gattii*, cause nearly all cryptococcal infections (Iyer et al., 2021). *C. neoformans* is an environmentally ubiquitous yeast, and under certain conditions, it can cause lethal meningoencephalitis in humans after triggering pulmonary infection in the lung due to inhalation of infectious particles (Normile et al., 2020). Like *C. albicans*, *C. neoformans* can form flower-like biofilms on abiotic surfaces (i.e., medical devices and coverslips surfaces), including ventriculoarterial shunt catheters, peritoneal dialysis fistulae, cardiac valves, and prosthetic joints (Martinez and Casadevall, 2015; Lopes et al., 2017). Moreover, on biotic surfaces, followed by traversing the blood–brain barrier in meningoencephalitis, *C. neoformans* also has the ability to form biofilm-like structures known as *Cryptococcomas* (Aslanyan et al., 2017). The biofilm formation of *C. neoformans* begins with the fungal adhesion to surfaces mediated mainly by glucuronoxylomannan (Chung and Brown, 2020). Furthermore, the glucuronoxylomannan, a major virulence factor synthesized intracellularly and then delivered to the extracellular space *via* vesicle-mediated secretion for assembly into the *C. neoformans* capsule, is also a component of the biofilm matrix of *C. neoformans* (Rizzo et al., 2020).

Biofilms are dynamic assemblies of microbial communities irreversibly attached to abiotic and biotic surfaces that are embedded in an exopolymeric matrix (Flemming et al., 2016). Biofilm is the common mode of microbial growth in natural environments and confers enhanced resistance of cells inside biofilms to treatment with conventional antimicrobials and immune responses compared with their planktonic counterparts (Ciofu et al., 2022). This highlights the fact that biofilm cells (i.e., cells within biofilms) develop the resistance to antimicrobial agents at concentrations 10–1,000 times higher when compared with those of planktonic cells (Jefferson et al., 2005). Furthermore, according to the National Institutes of Health survey, biofilms have been implicated in 65% of all microbial infections and 80% of all chronic infections, respectively (Jamal et al., 2018). Therefore, biofilms have been increasingly recognized as a highly attractive target for antimicrobial agents to combat biofilm-related infection. Currently, only a few antifungal agents, such as echinocandins and liposomal formulations of amphotericin B, are available for the treatment of such biofilm-based fungal infections, and multidrug resistance greatly hampers patient management (Kuhn et al., 2002; Marcos-Zambrano et al., 2016). Consequently, the development of novel antifungal agents that show promise against fungal biofilms is vital due to the increased incidence and mortality of invasive fungal infections.

The increasing incidence of drug-resistant fungi has attracted much attention from the scientific community towards studies on the potential antifungal activity of plant-derived natural substances (Stan et al., 2021), which have been widely employed in traditional medicine in some countries. Moutan cortex Radicis (MC), the root cortex of *Paeonia suffruticosa* Andrews, has been widely employed as an analgesic, antipyretic, and anti-inflammatory agent in traditional Chinese medicine (TCM) (Lin et al., 1999; Tatsumi et al., 2004), which can eliminate heat, promote blood flow, and remove blood stasis for the treatment of human diseases. Paeonol (20-hydroxy-40-methoxyacetophenone), a major phenolic component of MC, has been reported to possess excellent pharmacological and therapeutic activities (Li et al., 2009; Liu et al., 2017; Zhang et al., 2019; Vellasamy et al., 2021; Yang et al., 2021). For instance, paeonol injection has been effectively used in China for the treatment of inflammation and pain (Shanghai First Pharmaceutical Factory, 1973; Zhang et al., 2019) for approximately 50 years. Moreover, the pharmacokinetic properties of paeonol show that paeonol is quickly absorbed, extensively metabolized, and widely distributed in different tissues, such as the liver, heart, spleen, kidney, lung, and brain, without long-term accumulation in rats after oral administration (Hu et al., 2020). Currently, various dosage forms of paeonol have been explored and approved for use by China Food and Drug Administration, including tablets, injections, and external preparations such as ointments and adhesive plaster (http://app1.sfda.gov.cn/datasearchcnda/face3/dir.html). Paeonol exhibits robust anti-inflammatory activities; however, until now the antifungal and antibiofilm efficacy of paeonol against *C. albicans* and *C. neoformans* mono- and dual-species remain largely unexplored. The present study was aimed at assessing the antifungal and antibiofilm efficacy of paeonol, as well as the potential molecular mechanism underlying antibiofilm action against *C. albicans* and *C. neoformans*.

## Materials and Methods

### Reagents

Paeonol (purity ≥98%) was purchased from Chengdu Pulis Biological Science and Technology Co. Ltd. (Chengdu, China). Stock solutions of paeonol were dissolved in ddH_2_O at final concentrations of 5 mg ml^−1^ and kept at −20°C. SYTO 9, FUN^®^ 1, Calcofluor^®^ White M2R stain (CWS), Film Tracer SYPRO Ruby (SYPRO Ruby), wheat germ agglutinin (WGA), propidium iodide (PI), and 4′,6-diamidino-2-phenylindole (DAPI) dye were purchased from Invitrogen (Thermo Fisher Scientific, Waltham, Massachusetts, USA). Gatifloxacin was obtained from Shanghai Yuanye Bio-Technology Co. Ltd. (Shanghai, China). Other chemicals used were of analytical grade or better.

### Strain and Cultural Conditions

The single colonies of *C. albicans* SC5314 and *C. neoformans* H99 were picked up and routinely cultured in yeast peptone dextrose (YPD, Sigma-Aldrich, USA) plates at 30°C with shaking at 150 rpm. Overnight culture fungal stocks were prepared by mixing with an equal volume of 50% glycerol and storing at −80°C. Single and dual-species biofilms formed by *C. albicans* SC5314 and *C. neoformans* H99 were prepared in 96-well and 24-well plates (Costar, Corning, NY), respectively. For the preparation of dual-species biofilms, each fungal strain was grown to an OD_600_ of 0.5, and then equal volumes of each strain culture were mixed and added to each well to a final volume of 200 μl and 1 ml, respectively, and incubated at 30°C for 48 h.

A wild-type *Caenorhabditis elegans* (*C. elegans*) worm, which was gifted from Dr. Dan Xu, was employed in all assays and cultured in media supplemented with the chemical 5-fluoro-29-deoxyuridine (FUDR), which can prevent progeny from hatching. As previously described, nematodes of *C. elegans* were maintained and propagated on nematode growth medium (NGM) supplemented with the addition of *Escherichia coli* HB101 as a source of food. *C. albicans* SC5314 and *C. neoformans* H99 have been reported to be virulent against *C. elegans* (Mylonakis et al., 2002; Pukkila-Worley et al., 2011). *C. albicans* SC5314 and *C. neoformans* H99 were cultured in a liquid yeast extract-peptone-dextrose (YPD) broth or on brain heart infusion (BHI, Difco) agar supplemented with 45 mg ml^−1^ of kanamycin and stored at 30°C. M9 buffer was composed of 30% BHI supplemented with kanamycin (90 mg ml^−1^), ampicillin (200 mg ml^−1^), and streptomycin (200 mg ml^−1^) and was used in washing the worms, as previously reported (Tampakakis et al., 2008).

### Determination of Minimum Inhibitory Concentrations

The minimum inhibitory concentrations (MICs) of paeonol were measured for *C. albicans* SC5314 and *C. neoformans* H99 based on the broth microdilution method with a few modifications (Fu et al., 2021). Briefly, overnight cultures of *C. albicans* SC5314 and *C. neoformans* H99 were diluted with fresh RPMI-1640 (Sigma) or YPD medium, respectively. Subsequently, 100 μl of *C. albicans* SC5314 or *C. neoformans* H99 were transferred into individual wells of the 96-well microplate with final concentrations of 1 × 10^3^ and 10^5^ colony-forming units (CFUs) ml^−1^, respectively, and then paeonol was added to each well at final concentrations ranging from 0 to 1,000 μg ml^−1^ for a final volume of 200 μl per well. Fungal cells exposed to RPMI-1640 or YPD were employed as the negative control group. After the 96-well microplate was incubated at 30°C for 22–24 h, and for each culture, the optical density (OD) at 600 nm was examined using a spectrophotometer. Furthermore, the resulting fungal cultures were serially diluted, plated onto YPD agar, and further cultured for 24 h at 30°C to count the CFUs. The MIC was defined as the lowest concentration of paeonol that can inhibit the visible growth of the strain tested.

### Growth Kinetics of Fungal Cells Treated With Paeonol

The growth kinetics of paeonol against fungal cells were performed. Overnight-grown fungal cells were diluted in a fresh YPD medium and then incubated to a concentration of 1 × 10^6^ CFUs ml^−1^ at 30°C. The resulting fungal cell cultures with a final concentration of approximately 1 × 10^6^ CFUs ml^−1^ were added to each well of the 24-well plate. Subsequently, paeonol was added to each well for a final concentration of MIC and a volume of 600 μl and then further cultured for 24 h at 30°C. For CFU counts, 5 μl cultures were collected from each sample at predetermined time points (0, 2, 4, 6, 8, and 24 h), and the 10-fold dilutions of samples were incubated on YPD agar at 30°C. After 24 h of incubation, the number of CFU was determined.

### Morphological and Membrane Integrity Analysis of Paeonol-Treated Fungal Cells

Field emission scanning electron microscope (FESEM) analysis was used to examine the morphological changes of *C. albicans* SC5314 and *C. neoformans* H99 after exposure to paeonol. The fungal cell samples were prepared for FESEM analysis according to the method described in our previous report (Qian et al., 2020). Briefly, the fresh inoculum with final concentrations of approximately 1 × 10^6^ CFUs ml^−1^ was exposed to paeonol at different concentrations (0, MIC, and 2 MIC) at 30°C for 6 h. Paeonol-treated and paeonol-untreated cells were pelleted at 3,000 rpm for 10 min at 4°C, washed using phosphate-buffered saline (10 mM PBS), and prefixed with 2.5% glutaraldehyde at 4°C for 4 h. The prefixed cells were serially dehydrated using 30%, 50%, 70%, 80%, 90%, and 100% ethanol. Subsequently, the resulting cells were transferred into isoamyl acetate at 25°C for 1 h. Finally, the fixed cells were sputter-coated using an ion sputter, and the morphology of both the treated and untreated samples was imaged using a FESEM (Nova Nano SEM-450, FEI, Hillsboro, USA).

The cell membrane integrity of both *C. albicans* SC5314 and *C. neoformans* H99 mono- and dual-species cultures was respectively investigated using confocal laser scanning microscopy (CLSM; Zeiss LSM 880 with Airyscan), as previously described with slight modifications (Fu et al., 2021). Cell populations in logarithmic growth were treated with different concentrations of 0, MIC, and 2 MIC paeonol for 4 h, respectively. The resulting samples were then collected by centrifugation at 3,000 rpm for 10 min and resuspended in 10 mM PBS (pH 7.0). To visualize the cells using CLSM, a mixture of 2.5 μM SYTO 9 and 5 μM PI was thoroughly transferred into each cell suspension. After incubation at 25°C for 15–20 min, the samples were evaluated by CLSM, where the fluorescence emission spectra were used at excitation/emission wavelengths of 488/520 nm for SYTO 9 and 535/617 nm for PI, respectively.

### Analysis of Metabolic Activity of Fungal Cells Treated With Paeonol

The metabolic activities of both *C. albicans* SC5314 and *C. neoformans* H99 mono- and dual-species exposed to paeonol were examined using CLSM, as previously described (Qian et al., 2020). Mono- and dual-species cultures (approximately 1 × 10^6^ CFUs ml^−1^) of *C. albicans* SC5314 and *C. neoformans* H99 were treated with various concentrations of 0, MIC, and 2 MIC paeonol at 30°C for 24 h. The resulting cells were collected and washed gently three times with 10 mM PBS. After washing, the fungal cells were incubated with a mixture of 20 μM FUN^®^ 1 and 3 μM CWS. The cells were investigated *via* CLSM, followed by incubation for 45 min at 30°C, and with excitation/emission wavelengths of 470/590 nm for FUN^®^ 1 and 488/617 nm for CWS, respectively.

### Assessment of Gatifloxacin Influx to Paeonol-Treated Fungal Cells

To assess the cell wall damage of paeonol-treated mono- and dual-species suspensions of *C. albicans* SC5314 and *C. neoformans* H99, the characteristics of gatifloxacin transport across the fungal cell integrity barrier were investigated using CLSM, where gatifloxacin was used as a model antibiotic due to its intrinsic fluorescence (Qian et al., 2020). After mono- and dual-species of *C. albicans* SC5314 and *C. neoformans* H99 were cultivated overnight at 30°C, the cultures were diluted with fresh YPD to obtain an OD_600_ of 0.5. The resulting cell dilutions were then exposed to different concentrations of 0, MIC, and 2 MIC paeonol and further cultured at 30°C for 24 h. After the treated cells were washed three times with 10 mM PBS, 80 μg ml^−1^ gatifloxacin was then added, and the cells were incubated for another 6 h at 30°C. To visualize gatifloxacin influx to paeonol-treated cells, the cells were stained with 5 μM SYTO 9 at 25°C for 20 min. After incubation, the cells were rinsed three times with 10 mM PBS to remove redundant gatifloxacin and then observed using CLSM.

### Adhesion Assay of Fungal Cell to Abiotic Surfaces

For the adhesion assay, overnight cultures of *C. albicans* SC5314 and *C. neoformans* H99 were diluted with YPD to 1 × 10^6^ CFUs ml^−1^. A total of 900 μl of each cell dilutions were added to each well containing a sterilized glass coverslip of the 24-well plate and treated with various concentrations of 0, 1/4 MIC, 1/2 MIC, and MIC paeonol without shaking at 30°C for 4 h. After treatment, cell supernatants of each well were discarded, and nonadherent cells were removed by gently washing the coverslips 3 times with 10 mM PBS. Finally, the coverslips were immersed in the mixture of FUN^®^ 1 (5 μM) and CWS (5 μM), and the adhesion of fungal cells to the surface of the coverslip was evaluated using CLSM.

### Evaluation of Biofilm Formation by Paeonol-Treated *C. albicans* and *C. neoformans*

The effect of paeonol on the biofilm formation of *C. albicans* SC5314 and *C. neoformans* H99 mono- and dual-species was evaluated by FESEM and CLSM, as previously described (Qian et al., 2020; Qian et al., 2021). Briefly, mono- and dual-species cultures (1 × 10^6^ CFUs ml^−1^) of *C. albicans* SC5314 and *C. neoformans* H99 were cultured in each well of the 24-well plate containing a sterilized glass coverslip and treated with various concentrations of 0, 1/4 MIC, 1/2 MIC, and MIC paeonol at 30°C for 48 h. For biofilm evaluation by FESEM, 48-h biofilms formed by *C. albicans* SC5314 and *C. neoformans* H99 mono- and dual-species on the glass coverslips were fixed in 2.5% glutaraldehyde (v v^−1^) immediately at −4°C for 2 h followed by treatment and washed gently three times with 10 mM PBS. The fixed cells were then gradually dehydrated by rinsing for 10 min at each concentration using ascending grades of ethanol (30%, 50%, 70%, 90%, and 100%). Finally, the biofilm samples were inspected using FESEM. For examination using CLSM, biofilms were generated as described above. The biofilms were carefully rinsed three times with 10 mM PBS to remove nonadherent fungal cells. The biofilms were incubated with SYTO 9 for 15 min at 25°C. The biofilms were then rinsed twice with 10 mM PBS and observed under CLSM. For biofilm examination using an optical microscope, the biofilms were washed twice with 10 mM PBS to remove the planktonic cells and then stained with 0.1% (w v^−1^) crystal violet (CV). The samples were then washed three times using 10 mM PBS to remove redundant dyes. Finally, the biofilms were investigated by the optical microscope at ×400 magnification. For determination of the biofilm biomass, 150 μl of overnight cultures (approximately 1 × 10^6^ CFUs ml^−1^) of mono- and dual-species were added to each well of the 96-well plate and exposed to paeonol at 0, 1/4 MIC, 1/2 MIC, and MIC for 48 h at 30°C. After incubation, the culture medium was removed, and the biofilms were then rinsed twice with 10 mM PBS and stained with 0.1% CV (w v^−1^) for 20 min at 25°C. Finally, the OD_570_ of each well was measured using a microplate reader.

### Effect of Paeonol on Preformed Biofilms by *C. albicans* and *C. neoformans*

The damage effect of paeonol on cells within preformed biofilms was evaluated using CLSM based on our previous method (Qian et al., 2020; Qian et al., 2021). Briefly, mono- and dual-species cultures (approximately 1 × 10^6^ CFUs ml^−1^) of *C. albicans* SC5314 and *C. neoformans* H99 were cultured in individual wells of a 24-well plate for 48 h at 30°C, with glass coverslips in each well. The resulting preformed biofilms were treated with various concentrations of 0, 4 MIC, 8 MIC, or 16 MIC paeonol for 6 h at 30°C. The biofilms were then washed twice with the addition of 10 mM PBS to remove planktonic cells and stained with a mixture of SYTO 9 and PI dyes. After incubation for 20 min, viable and nonviable cells within preformed biofilms were observed using CLSM.

The dispersion capability of paeonol against preformed biofilms by mono- and dual-species of *C. albicans* SC5314 and *C. neoformans* H99 was evaluated quantitatively and qualitatively using the CV assay, an optical microscope, and FESEM. Mono- and dual-species suspensions (1 × 10^6^ CFUs ml^−1^) of *C. albicans* SC5314 and *C. neoformans* H99 cells were added into each well of the 24-well microtiter plate containing glass coverslips. The resulting 48-h preformed biofilms were exposed to four concentrations of 0, 4 MIC, 8 MIC, and 16 MIC paeonol for 8 h at 30°C. For qualitative analysis, the biofilms were examined using an optical microscope and FESEM. Similarly, for quantitative analysis, the biofilm biomass was determined using the CV assay, as described above.

### Analysis of Virulence Phenotypes of *C. albicans* and *C. neoformans* Treated With Paeonol

To assess the inhibition effect of paeonol on the hyphal formation of *C. albicans* SC5314, *C. albicans* SC5314 was grown in YPD supplemented with 10% fetal bovine serum (FBS) (Thermo Fischer, Belgium) to induce the switch from budding to hyphal growth of *C. albicans* SC5314 while incubated with various concentrations of paeonol (0, 1/2 MIC, MIC, 2 MIC). After 24 h of incubation, yeast-like cells and/or hyphae were assessed microscopically. Moreover, to examine the effect of paeonol on the capsule production of *C. neoformans* H99, the assay was carried out as previously described (Nichols, 2021). Briefly, the *C. neoformans* H99 (approximately 1 × 10^6^ CFUs ml^−1^) was treated with various concentrations of paeonol (0, 1/2 MIC, MIC, 2 MIC) at 30°C for 24 h. Fungal cells were then collected and washed three times with 10 mM PBS, resuspended in Dulbecco’s modified Eagle’s medium (DMEM; GIBCO, USA), and further cultured for 48 h at 30°C. Finally, 0.1 ml of the fungal culture was mixed with an equal volume of India ink (Phygene, China), and the capsule was visualized under the microscope. Similarly, to evaluate the effect of paeonol on melanin production in *C. neoformans* H99, the assay was conducted as previously described (Nichols, 2021). A total of 5 µl of *C. neoformans* H99 cells (approximately 1 × 10^6^ CFUs ml^−1^) were spotted on melanin-inducing minimal medium (15 mM glucose, 10 mM MgSO_4_, 21.4 mM KH_2_PO_4_, 13 mM glycine, 3 µM thiamine, 1.5% (w v^−1^) Bacto agar, pH 5.5) supplemented with 1 mM l-DOPA and different concentrations of paeonol (0, 1/4 MIC, 1/2 MIC, and MIC) and further cultured at 30°C for 48 h at 150 rpm and protected from light. The melanin production was visually examined and photographed.

### Effect of Paeonol on Lifespan of *C. elegans* and *C. elegans* Infected With Fungal Cells

The examination of *C. elegans* lifespan was conducted as previously reported (Apfeld and Kenyon, 1999). Paeonol was added to standard NGM plates to assess the potential toxicity. The chemical FUDR (75 μM) was employed on prefertile young-adult nematodes to prevent their progeny from growth unless stated otherwise. Moreover, the infection assay was performed as previously reported (Okoli et al., 2009). Briefly, *C. elegans* N2 nematodes grown on NGM plates were washed with M9 buffer, transferred into *C. albicans* SC5314 or *C. neoformans* H99 lawns that were cultured for 48 h at 30°C on YPD, and inoculated for another 6 h of coculture. The *C. elegans* N2 worms infected by *C. albicans* SC5314 or *C. neoformans* H99 were then washed twice with M9 buffer, transferred into NGM plates supplemented with paeonol at various concentrations (0, MIC, and 2 MIC), and further cultured for 24 h. After treatment, the N2 worms were collected by washing off the plates using M9 buffer. Subsequently, 20 N2 worms were transferred into each well of a 24-well plate. Plates were sealed with Breathe-Easy™ membranes and incubated for 20 days at 20°C. The worm survival rate was examined every 2 days using a dissecting microscope, and the survival rates were analyzed. The experiments were performed in triplicate.

### RNA Sequencing and Data Analysis

RNA sequencing was used to identify genes regulated by paeonol treatment. For the preparation of paeonol-treated or paeonol-untreated *C. albicans* SC5314 and *C. neoformans* H99 biofilm cells, fungal cells (approximately 1 × 10^6^ CFUs ml^−1^) were seeded into a 90-mm sterile petri dish containing 5 ml of YPD medium, and was cultured statically for 1 h to allow initial adherence of fungal cells. A total of 15 ml of fresh YPD medium supplemented with or without 1/2 MIC paeonol was added to obtain a final volume of 20 ml. Here, 1/2 MIC paeonol was employed to ensure the inhibitory effect on biofilm formation of fungal cells and obtain a sufficient number of cells to allow RNA extraction. The dishes were then incubated with shaking at 50 rpm for 48 h at 30°C. Subsequently, the dishes were rinsed gently three times with 10 mM PBS to remove unattached cells. Biofilm cells were then detached by gently scraping with a sterile scraper, followed by centrifugation and resuspension. Finally, cell pellets were stored at −80°C.

Total RNAs from biofilm cells were extracted using RNAiso Plus (Takara, Dalian, China) according to the manufacturer’s instructions. RNA concentration and quality were measured using a NanoDrop 2000 (Thermo Fisher Scientific, Wilmington, USA), and cDNA libraries were constructed using NEBNext RUltraTM RNA Library Prep Kit (NEB, USA) following the manufacturer’s recommendations. The cDNA libraries were then sequenced using a Novoseq sequencer (Illumina, USA) to achieve 150 bp paired-end reads. Clean reads from RNA-sequencing raw data were assembled into full-length transcriptomes with the reference genomes of *C. albicans* (http://www.candidagenome.org) and *C. neoformans* (https://fungidb.org/fungidb/app). Differential gene expression (DEG) analysis between treated and untreated samples was performed using the Bioconductor software package DESeq2 in R 1.16.1 (Love et al., 2014). Fragments per kilobase of transcript sequence per million base pairs sequenced (FPKM) and log2FoldChange were applied to evaluate relative gene expression. *p*-values were adjusted to generate false discovery rates (padj) with the significance threshold for DEGs as padj <0.05 (Benjamini and Yekutieli, 2001).

### Quantitative Real-Time Reverse Transcription PCR

The extraction of total RNAs and quantitative real-time reverse transcription PCR (qRT-PCR) were carried out using the Total RNA Isolation Kit and ChamQ Universal SYBR qPCR Master Mix according to the manufacturer’s instructions (Vazyme Biotech Co. Ltd., Nanjing, China), respectively. All the primers were synthesized by Sangon Biotech Co. Ltd. (Shanghai, China), and their oligonucleotide sequences are displayed in **Supplementary Table S1**. A relative quantification method (2^−ΔΔCT^) was employed to examine the changes in expression following paeonol treatment.

### Statistical Analysis

Experiments were conducted independently in triplicate. For the multiple-group comparisons, statistical analysis was carried out using SPSS software (SPSS 8.0 for Windows). An analysis of variance was performed to determine any significant differences (*p* ≤ 0.01).

## Results

### MIC_S_ and Growth Kinetics of Paeonol for *C. albicans* SC5314 and *C. neoformans* H99

**Figure 1:**
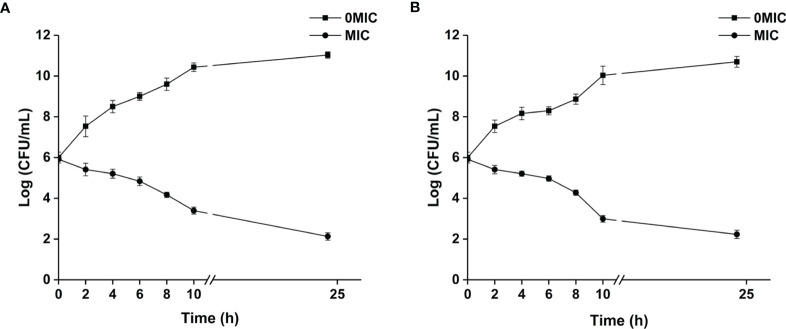
The growth kinetics of *C. albicans* SC5314 **(A)** and *C. neoformans* H99 **(B)** in response to paeonol were determined. The growth kinetics of fungal cells in the presence and absence of paeonol at an MIC concentration were plotted. All data were expressed as means ± SD (*n* = 3).

The MIC value of paeonol for *C. albicans* SC5314 and *C. neoformans* H99 was determined. The results demonstrated that MICs of paeonol for single-species cultures of *C. albicans* SC5314 and *C. neoformans* H99 were 250 μg ml^−1^, and the MIC of paeonol for mixed-species cultures was 500 μg ml^−1^. The results indicated that paeonol exhibited potent antifungal efficacy against single- and mixed-species cultures of *C. albicans* SC5314 and *C. neoformans* H99. The growth kinetics of fungal cells exposed to paeonol was monitored by measuring OD at 600 nm at various time intervals. As demonstrated in **Figure 1**, paeonol effectively inhibited the growth of *C. albicans* SC5314 and *C. neoformans* H99 within 24 h of incubation compared with the untreated control group, suggesting that paeonol possessed robust antifungal activities against *C. albicans* SC5314 and *C. neoformans* H99.

### Paeonol Exposure Damaged Cell Membrane Integrity, Lowered Metabolic Activities, and Altered Cell Morphology of *C. albicans* SC5314 and *C. neoformans* H99

**Figure 2:**
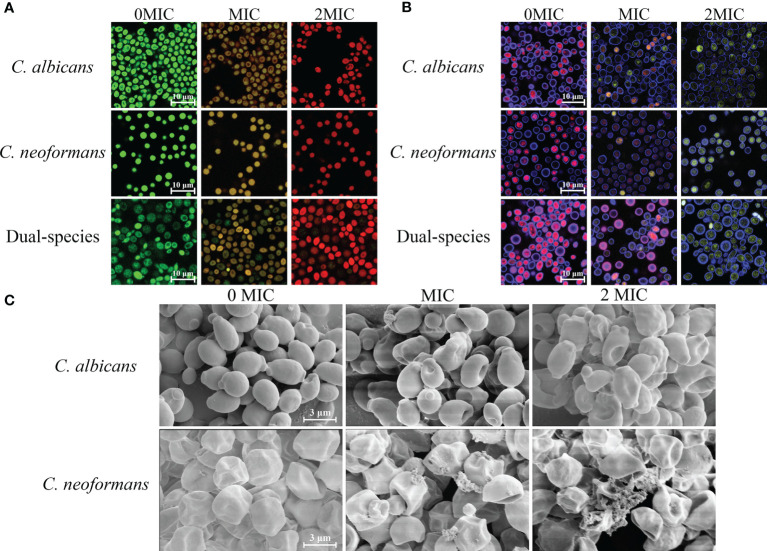
Effects of paeonol on the cell membrane integrity **(A)**, metabolic activities **(B)**, and cell morphology **(C)** of *C. albicans* SC5314 and *C. neoformans* H99 mono- and dual-species. **(A)** *C. albicans* SC5314, *C. neoformans* H99, and dual-species cells exposed to paeonol at 0, MIC, and 2 MIC at 30°C for 4 h were shown using a confocal laser scanning microscope (CLSM) in combination with SYTO 9 and propidium iodide. **(B)** After *C. albicans* SC5314, *C. neoformans* H99 mono- and dual-species were incubated with paeonol at 0, MIC, and 2 MIC for 24 h at 30°C; images were captured using CLSM by combining FUN^®^ 1/Calcofluor^®^ White M2R dyes. **(C)** *C. albicans* SC5314 and *C. neoformans* H99 were exposed to paeonol at different concentrations of 0, MIC, and 2 MIC at 30°C for 6 h and assessed using field emission scanning electron microscope (FESEM). Scale bars represent 10 and 3 μm for CLSM and FESEM images, respectively.

SYTO 9 and PI were employed as fluorescent dyes to evaluate cell membrane integrity, and fungal cells with intact cell membranes stain fluorescent green, whereas fungal cells with compromised membranes stain fluorescent red. Therefore, the integrity of the fungal cell membrane was examined by staining with SYTO 9/PI. As demonstrated in **Figure 2A**, CLSM images displayed obvious changes in cell membrane integrity of mono- and dual-species cultures of *C. albicans* SC5314 and *C. neoformans* H99 exposed to paeonol at two concentrations of MIC and 2 MIC compared with those of the untreated group. The bright green fluorescence was observed in the untreated group, suggesting that the cell membrane integrity was intact, whereas the red/green fluorescence ratios showed an increase with enhanced paeonol concentrations. Overall, these findings indicate that paeonol exposure disrupted cell membrane integrity of mono- and dual-species cultures of *C. albicans* SC5314 and *C. neoformans* H99 in a dose-dependent manner.

The staining procedure involves the addition of FUN^®^ 1 and CWS. FUN^®^ 1 discriminates between metabolically active and metabolically inactive or dead fungal cells and produces red-fluorescent cylindrical intravacuolar structures and diffuse green-yellow fluorescence, respectively. In addition, CWS can bind to chitin and cellulose contained in cell walls in fungal cells, staining them fluorescent blue. As shown in **Figure 2B**, almost no red-to-yellow-green fluorescence conversion was observed in the untreated mono- and dual-species cells of *C. albicans* SC5314 and *C. neoformans* H99. By contrast, in the presence of paeonol at MIC or 2 MIC, the area with red fluorescence was clearly reduced in the mono- and dual-species *C. albicans* SC5314 and *C. neoformans* H99 cultures compared with that of the untreated control group. By contrast, after exposure to paeonol at the MIC, fewer red fluorescence areas were observed when compared with the untreated control group. High-concentration paeonol treatment increased the conversion from red to yellow-green fluorescence, suggesting that *C. albicans* SC5314 and *C. neoformans* H99 cells in the mono- and dual-species cultures exhibited low-level metabolic activity when treated with paeonol.

As shown in **Figure 2C**, FESEM images clearly showed cell surface bulging and damaged cell structure of *C. albicans* SC5314 due to the treatment of paeonol. Similarly, FESEM results demonstrated that paeonol had a morbid effect on *C. neoformans* H99 cells’ surface. After treatment with paeonol, the surface of the fungal cells became seriously corrugated, while the paeonol-untreated cells presented a bright and smooth surface. Moreover, the enhanced extent of destruction due to the increased concentration of paeonol was clearly revealed based on the severely distorted cell structures of *C. albicans* SC5314 and *C. neoformans* H99 exposed to 2 MIC paeonol.

### Paeonol Treatment Increased Gatifloxacin Influx to *C. albicans* SC5314 and *C. neoformans* H99 Mono- and Dual-Species Cells

**Figure 3:**
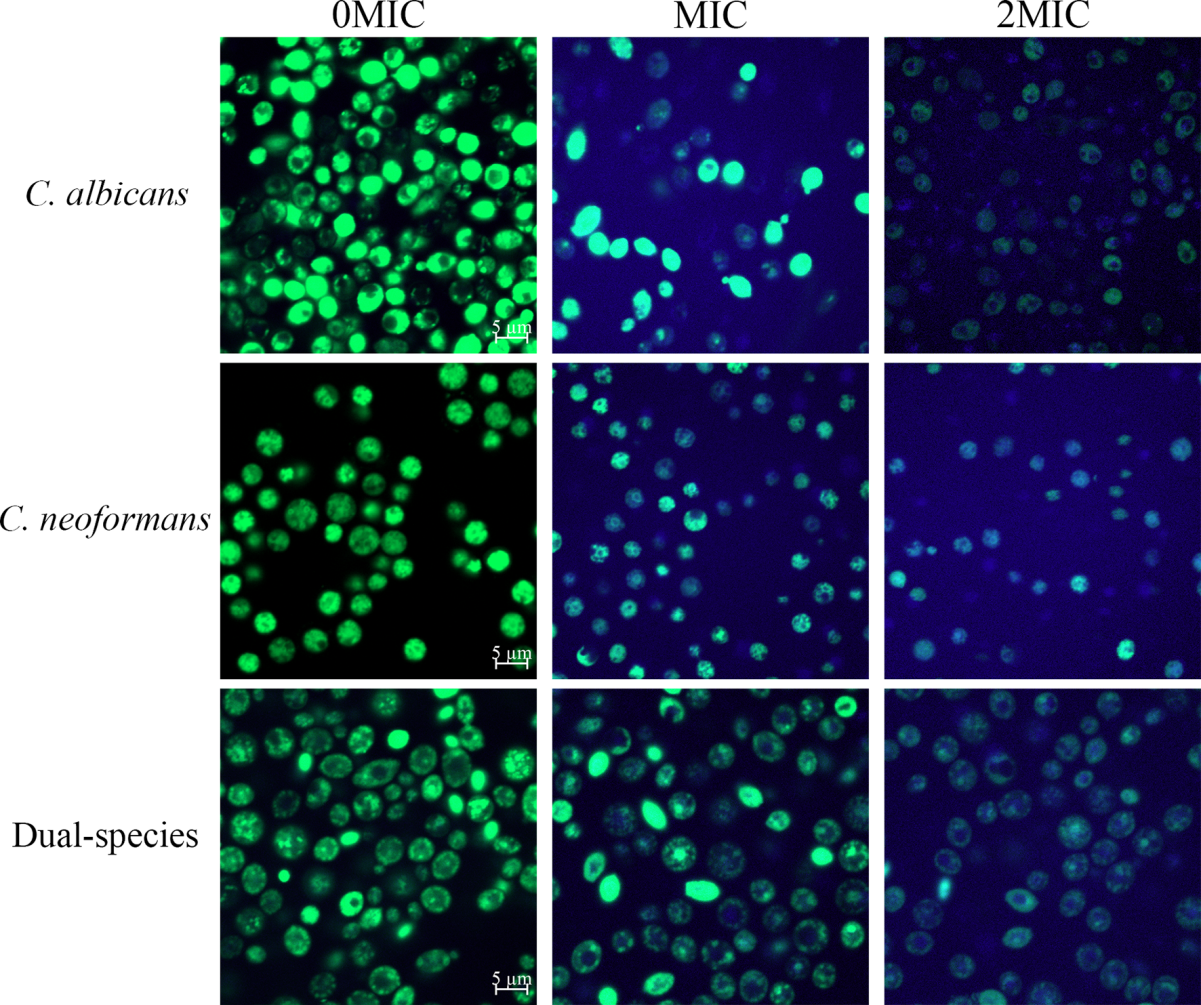
Examination of gatifloxacin influx to cells of *C. albicans* SC5314 and *C. neoformans* H99 mono- and dual-species cultures following exposure to paeonol by confocal laser scanning microscope combined with STYO 9 (green fluorescent signals) and gatifloxacin (blue fluorescent signals) with its intrinsic fluorescence characteristics. Fluorescent signals were generated in fungal cells cultured in YPD medium supplemented with final concentrations of 0, MIC, and 2 MIC. Scale bars represent 5 μm.

To examine the effect of paeonol on the cell wall integrity, STYO 9 and gatifloxacin with the intrinsic blue fluorescence were employed using CLSM. As shown in **Figure 3**, untreated cells emitted strong green fluorescence, suggesting that the integrity of the cell wall was intact. In contrast, treating mono- and dual-species cultures of *C. albicans* SC5314 and *C. neoformans* H99 with paeonol at MIC or 2 MIC yielded more blue fluorescence compared with those of the untreated cells. These results showed that paeonol treatment enhanced the influx of gatifloxacin to fungal cells by disrupting the cell wall integrity of *C. albicans* SC5314 and *C. neoformans* H99 in both mono- and dual-species with an increase in the addition of paeonol concentration.

### Paeonol Exposure Inhibited the Adhesion of *C. albicans* SC5314 and *C. neoformans* H99 Mono- and Dual-Species Cells to the Abiotic Surface

**Figure 4:**
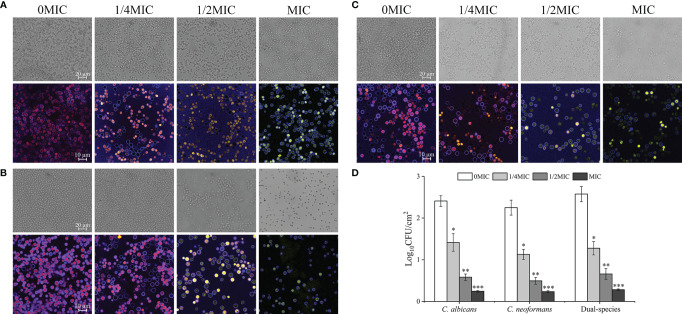
Effect of paeonol on the adhesion to an abiotic surface and metabolic activity of *C. albicans* SC5314, *C. neoformans* H99, and dual-species. **(A–D)** The changes of *C. albicans* SC5314 **(A)**, *C. neoformans* H99 **(B)**, and dual-species **(C)** adhesion were assessed using a confocal laser scanning microscope (CLSM) in bright-field mode and viable cell counts **(D)** when exposed to final concentrations of paeonol at 0, 1/4 MIC, 1/2 MIC, and MIC. **(A–C)** The metabolic activity of treated and untreated *C. albicans* SC5314 **(A)**, *C. neoformans* H99 **(B)**, and dual-species **(C)** were evaluated using CLSM in combination with FUN^®^ 1/Calcofluor^®^ White M2R dyes under fluorescence and reflection mode. Scale bars represent 20 μm for bright-field mode and 10 μm for fluorescence and reflection mode. Bars represent the standard deviation (*n* = 3). ^*^ *p* < 0.05; ^**^ *p* < 0.01; ^***^ *p* < 0.001.

The effect of paeonol on *C. albicans* SC5314 and *C. neoformans* H99 mono- and dual-species culture adhesion on the glass coverslips surface was examined with FUN^®^ 1 and CWS dyes using a CLSM. The images obtained after 4 h of mono- and dual-species coincubation with paeonol are displayed in **Figure 4**. Following treatment with various concentrations of paeonol, the number of adherent cells exhibited a gradual reduction trend compared with the untreated group. Furthermore, there was a decrease in the metabolic activity of fungal cells in the paeonol-treated group at 1/2 MIC or MIC. In particular, the MIC concentration of paeonol could significantly inhibit cell attachment of *C. albicans* SC5314 and *C. neoformans* H99 mono- and dual-species to the glass surface compared with the untreated group (^***^ *p* < 0.001) (**Figure 4D**). Moreover, fluorescence micrographs of the untreated or 1/4 MIC-treated mono- and dual-species cells of *C. albicans* SC5314 and *C. neoformans* H99 showed red fluorescence. In contrast, the mono- and dual-species *C. albicans* SC5314 and *C. neoformans* H99 cells in the presence of paeonol at 1/2 MIC or MIC were overlaid with yellow or green fluorescences. These findings indicate that paeonol at concentrations greater than 1/2 MIC inhibited *C. albicans* SC5314 and *C. neoformans* H99 mono- and dual-species cell adhesion to the glass coverslip surface by reducing their metabolic activities.

### Paeonol Exposure Interfered With Biofilm Formation With *C. albicans* SC5314 and *C. neoformans* H99 Mono- and Dual-Species Cells

**Figure 5:**
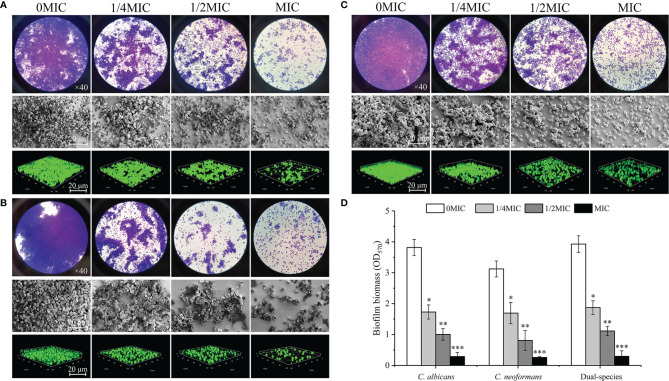
Examination of effects of paeonol on the biofilm formation of *C. albicans* SC5314 **(A)**, *C. neoformans* H99 **(B)**, and dual-species **(C)** with both qualitative and quantitative approaches. **(A–C)** Qualitative analysis of biofilm formation was examined at final concentrations of paeonol ranging from 0 to MIC using the light microscope (objective lens, ×40), field emission scanning electron microscope (FESEM), and confocal laser scanning microscope (CLSM). **(D)** Inhibitory effects of paeonol on biofilm formation were assessed quantitatively using the crystal violet staining assay. Scale bars represent 20 μm for FESEM and CLSM. Bars represent the standard deviation (*n* = 3). ^*^ *p* < 0.05; ^**^ *p* < 0.01; ^***^ *p* < 0.001.

The effect of paeonol treatment on biofilm formation by mono- and dual-species of *C. albicans* SC5314 and *C. neoformans* H99 was investigated using an optical microscope, FESEM, CLSM, and CV observations. There were a couple of purple patches on the images for untreated biofilms, as shown in **Figures 5A–C**, indicating that mono- and dual-species cells produced dense biofilms on the glass coverslips in the untreated group. In contrast, the biofilm biomass decreased slightly in mono-and dual-species *C. albicans* SC5314 and *C. neoformans* H99 when treated with exposure to paeonol at 1/4 MIC. In addition, when *C. albicans* SC5314 and *C. neoformans* H99 were treated with paeonol at MIC, a heterogeneous, negligible biofilm was observed, in which only a few scattered aggregates of mono-species were surveyed in a monolayer manner. Similarly, treatment with paeonol at 1/2 MIC or MIC restrained biofilm formation of dual-species with sparse clusters, and the aggregation propensity was lower than that of the untreated group. Moreover, **Figure 5D** shows that paeonol exposure inhibited biofilm formation of *C. albicans* SC5314 and *C. neoformans* H99 mono- and dual-species in a dose-dependent manner. There was a significant difference between the 1/2 MIC- or MIC-treated group and the untreated group. Collectively, these results indicated that paeonol could effectively restrain biofilm formation of *C. albicans* SC5314 and *C. neoformans* H99 mono- and dual-species.

### The Effect of Paeonol on Preformed Biofilms of *C. albicans* SC5314 and *C. neoformans* H99 Mono- and Dual-Species Cultures

**Figure 6:**
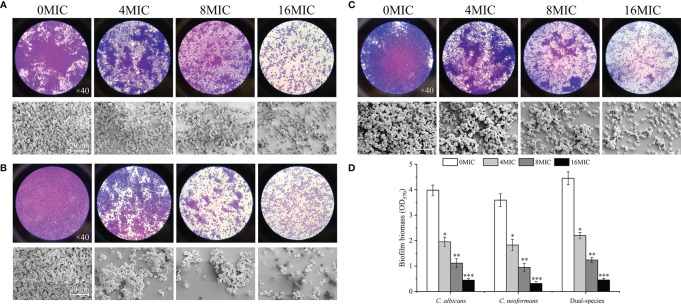
Dispersal effects of paeonol on the preformed biofilm of *C. albicans* SC5314 **(A)**, *C. neoformans* H99 **(B)**, and dual-species **(C)**. **(A–C)** Preformed biofilms treated with various final concentrations of paeonol ranging from 0 to 16 of the MIC were examined using the light microscope (objective lens, ×40) and field emission scanning electron microscope (FESEM). **(D)** The changes of paeonol-treated preformed biofilms were examined using the crystal violet staining assay. Scale bars represent 20 μm. Bars represent the standard deviation (*n* = 3). ^*^ *p* < 0.05; ^**^ *p* < 0.01; ^***^ *p* < 0.001.

The dispersal activity of paeonol on preformed biofilms formed by mono- and dual-species *C. albicans* SC5314 and *C. neoformans* H99 was examined by an optical microscope and FESEM. As shown in **Figures 6A–C**, mono- and dual-species in the untreated group formed dense and uniform biofilms, as evidenced by the presence of multilayer biofilms. Reductions of 72% and 74% were achieved for *C. albicans* SC5314 and *C. neoformans* H99 after 6 h of incubation with paeonol at 8 MIC. The highest decline in *C. albicans* SC5314 and *C. neoformans* H99 biofilm dispersal was achieved with paeonol at 16 MIC, with a decrease of almost 90% and 92%, respectively, and this was the most promising treatment concentration. Similarly, treatment with paeonol at 16 MIC was capable of triggering an almost complete dispersal of 48-h preformed biofilms by *C. albicans* SC5314 and *C. neoformans* H99 mono- and dual-species (**Figure 6D**). In addition, the results from the CFU count displayed a remarkable decline in viable biofilm cells of *C. albicans* SC5314 and *C. neoformans* H99 in a concentration-dependent manner when mono- and dual-species biofilms were exposed to paeonol (data unpublished).

**Figure 7:**
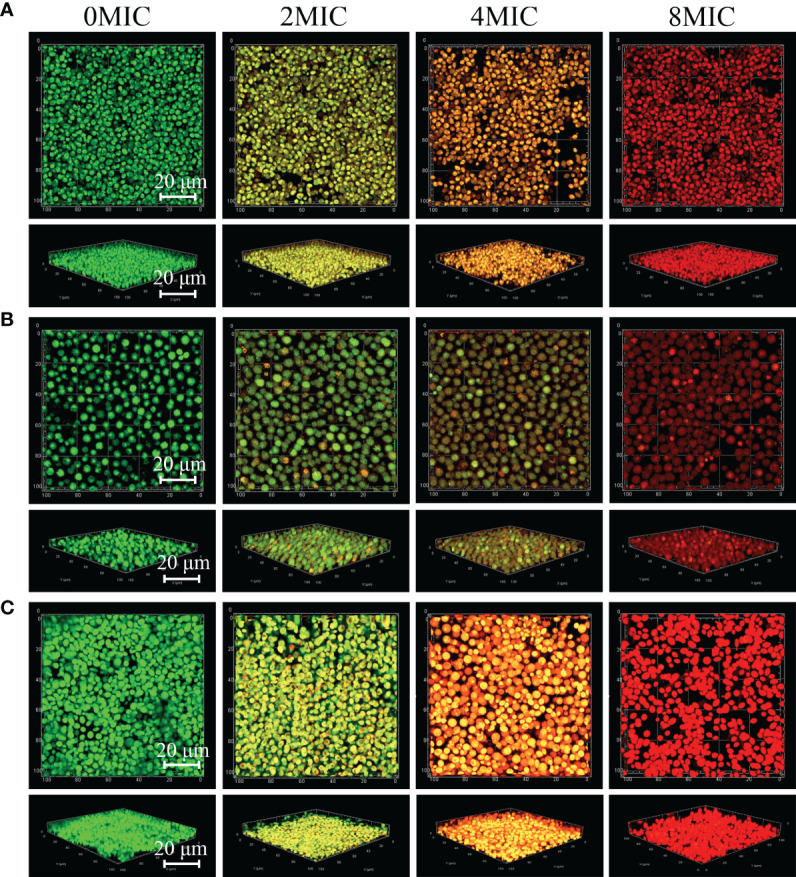
2D and 3D micrographs of viable and damaged biofilm cells of *C. albicans* SC5314 and *C. neoformans* H99 mono- and dual-species using confocal laser scanning microscope. *C. albicans* SC5314 **(A)**, *C. neoformans* H99 **(B)**, and dual-species **(C)** biofilm cells were treated with paeonol for 6 h at 0, 2 MIC, 4 MIC, and 8 MIC, and images were captured using a confocal laser scanning microscope in combination with SYTO 9 and propidium iodide. Viable and damaged biofilm cells emitted green and red fluorescence, respectively. Scale bars represent 20 μm.

Viable biofilm cells of *C. albicans* SC5314 and *C. neoformans* H99 mono- and dual-species were evaluated with SYTO 9, while damaged or nonviable biofilm cells were labeled with PI. In the presence of 2 MIC paeonol, a small number of nonviable cells exhibiting low yellow or red fluorescence were observed within mono- and dual-species biofilms that shifted deeper into the image stack, as shown in **Figure 7**. Similar to paeonol treatment at 2 MIC, yellow or red fluorescence emitted by nonviable cells increased in the presence of paeonol at 4 MIC. Furthermore, when the paeonol concentration was increased to 8 MIC or higher, the overwhelming majority of cells within single- and dual-species biofilms of *C. albicans* SC5314 and *C. neoformans* H99 emitted red fluorescence, indicating that paeonol damaged the membrane of biofilm cells in a dose-dependent manner.

### Paeonol Impaired the Virulence Factor of *C. albicans* SC5314 and *C. neoformans* H99

**Figure 8:**
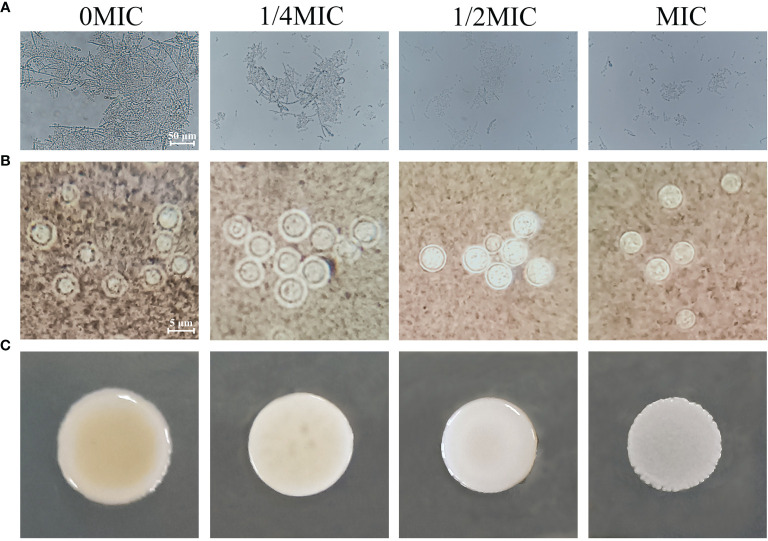
Effects of paeonol on the hyphal development of *C. albicans* SC5314 **(A)**, as well as the capsule **(B)** and melanin **(C)** production of *C. neoformans* H99. **(A)** *C. albicans* SC5314 was cultured on hypha-inducing medium supplemented with different concentrations of paeonol and imaged using a fluorescence-inverted microscope (scale bars, 50 μm). **(B)** India ink staining of the capsule of paeonol-treated *C. neoformans* H99. After *C. neoformans* H99 were treated with paeonol at 0, 1/4 MIC, 1/2 MIC, and MIC, the images were captured using the optical microscope (scale bars, 5 μm). The alterations of capsule thickness by paeonol-treated *C. neoformans* H99 were evaluated by measuring the capsule thickness of at least 30 cells. **(C)** Analysis of the melanin production of *C. neoformans* H99 exposed to paeonol. *C. neoformans* H99 was cultured on melanin-inducing medium supplemented with various concentrations of paeonol for 48 h at 30°C, and then examined.

*C. albicans* cells exist in various morphological states and switch from a budded or yeast-like form to a pseudohyphal or true hyphal filamentous form, which is associated with virulence. A microplate-based morphological assay was performed to elucidate the effect of paeonol on the hyphal development of *C. albicans* SC5314. As shown in **Figure 8A**, the microscopic examination revealed that the budded-to-hyphal-form transition of untreated *C. albicans* SC5314 cells was apparent and showed distinct hyphal elongation in the hypha-inducing medium. In contrast, the addition of MIC paeonol inhibited the budded-to-hyphal-form transition of *C. albicans* SC5314, and this inhibition was dose dependent. Moreover, the *C. neoformans* H99 capsule, mainly composed of glucuronoxylomannan, polysaccharides, and galactoxylomannan, is a major contributor to its virulence. In this study, India ink staining was applied to reveal changes in capsular thickness of *C. neoformans* H99 in the absence and presence of paeonol. As demonstrated in **Figure 8B**, the thicker capsules surrounding the untreated *C. neoformans* H99 cells were observed, whereas the capsule thickness of the MIC- or 2 MIC-treated group markedly decreased, particularly with the apparent loss of capsule thickness under 2 MIC paeonol-treated circumstances. Similarly, in *C. neoformans* H99, melanin represents the second major virulence factor after the presence of a capsular polysaccharide. To reveal the effect of paeonol on the melanin production of *C. neoformans* H99, the melanin production was measured using a melanin-inducing medium. As shown in **Figure 8C**, in the absence of paeonol, *C. neoformans* H99 produced abundant melanin, whereas a significant decline in melanin production was observed when *C. neoformans* H99 was treated with paeonol at 1/2 MIC. Taken together, our results indicate that paeonol could impair the effective capsule and melanin production of *C. neoformans* H99.

### Paeonol Prolonged *C. elegans* Lifespan and Protected *C. albicans* SC5314*-* and *C. neoformans* H99-Infected *C. elegans*

**Figure 9:**
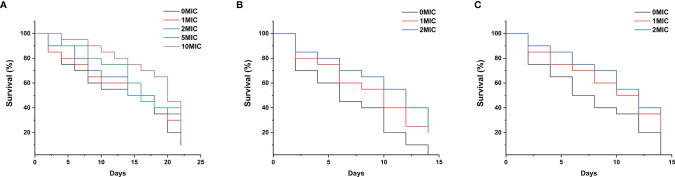
**(A)** The growth of *C. elegans* was determined in the presence of high concentrations of paeonol, and the survival rate was monitored in different treatment groups. **(B, C)** Survival of *C. elegans* exposed to the pathogenic fungi *C. albicans* SC5314 **(B)** and *C. neoformans* H99 **(C)** in the presence of different concentrations of paeonol. The survival rate of *C. elegans* was evaluated in the various treatment groups. The values refer to the mean survival rate of three biological replicates.

To assess the effect of paeonol on the *C. elegans* lifespan, the young fertile adults were treated with different concentrations of paeonol ranging from 0 to 10 MIC for 24 h. As shown in **Figure 9A**, the results indicated that paeonol did not shorten the lifespan of nematodes at the doses tested. Conversely, treatment with 5 MIC and 10 MIC paeonol significantly prolonged the lifespan of nematodes compared with the negative control group, suggesting that nematodes treated with paeonol may benefit from a high-dose diet. To examine the antifungal effect of paeonol against *C. albicans* SC5314 and *C. neoformans* H99 in *C. elegans*, the *C. elegans* exposed to *C. albicans* SC5314 or *C. neoformans* H99 were treated with different concentrations of paeonol for 24 h. As displayed in **Figures 9B, C**, the mean lifespan of *C. elegans* infected with *C. albicans* SC5314 and *C. neoformans* H99 was 6.9 ± 0.5 days and 7.1 ± 0.6 days, respectively. By contrast, when treated with 2 MIC paeonol, the average lifespan of *C. elegans* infected with *C. albicans* SC5314 and *C. neoformans* H99 was 10.1 ± 0.8 days and 11.7 ± 0.7 days, in which paeonol prolonged the mean lifespan of infected *C. elegans* by 46.38% and 64.79%, respectively. Thus, these data suggest that paeonol can effectively restrain the growth of *C. albicans* SC5314 or *C. neoformans* H99 in *C. elegans*, indicating that paeonol treatment effectively reverses the adverse effects of *C. albicans* SC5314 and *C. neoformans* H99 on nematodes, thus prolonging the lifespan of *C. elegans* during infection.

### Transcriptomic Profiling of *C. albicans* SC5314 and *C. neoformans* H99 Biofilm Cells Treated With Paeonol

**Figure 10:**
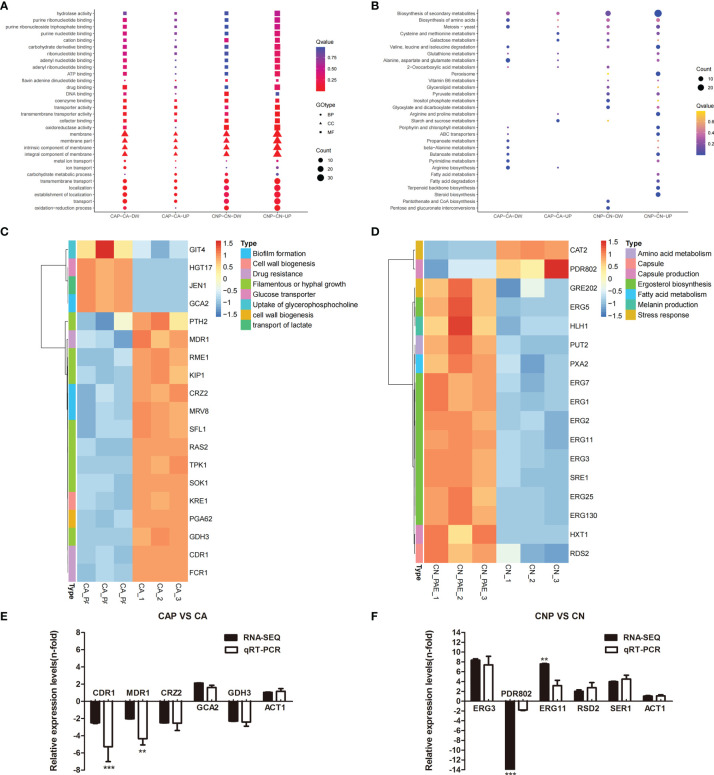
Transcriptomic analysis of paeonol-treated *C. albicans* SC5314 and *C. neoformans* H99 biofilms. **(A, B)** The Gene Ontology **(A)** and Kyoto Encyclopedia of Genes and Genomes **(B)** pathway enrichment analysis of differentially expressed genes (DEGs) in paeonol-treated *C. albicans* SC5314 (CAP) and *C. neoformans* H99 (CNP) biofilms as compared with the untreated control were depicted. After *C. albicans* SC5314 and *C. neoformans* H99 biofilms were exposed to paeonol at 1/2 MIC, respectively, their transcriptomic profiles were examined. The sphere size was proportional to the number of DEGs. **(C, D)** Heat maps of the DEGs related to *C. albicans* SC5314 **(C)** and *C. neoformans* H99 **(D)** biofilm formation or virulence factors were constructed using ImageGP, where absolute *n*‐fold changes of DEGs in three biological replicates in each condition, and their corresponding biological function on the right side of each panel were depicted. **(E, F)** The expression of selected DEGs and ACT1 (control) during *C. albicans* SC5314 **(E)** and *C. neoformans* H99 **(F)** biofilm formation in the presence of paeonol relative to their expression under the untreated condition was quantified by quantitative reverse transcription polymerase chain reaction (qRT-PCR, light grey bars) to compare RNA-sequencing data (RNA-SEQ, black bars). Bars represent the average relative change in RNA abundance of the indicated genes, and error bars indicate standard deviations (*n* = 3 in each condition). Asterisks depict significantly different gene expression levels as compared between RT-PCR and RNA-sequencing data using a standard Student’s *t*-test (^**^ *p* < 0.01; ^***^ *p* < 0.001).

To reveal the antibiofilm mechanism of paeonol at the molecular level, RNA-sequencing was employed to obtain further insight into alterations in gene expression profiles of *C. albicans* SC5314 and *C. neoformans* H99 biofilms following paeonol treatment. In paeonol-treated *C albicans* SC5314 biofilms, a total of 159 DEGs were found, among which 131 genes displayed a decrease in expression and only 28 genes demonstrated an increase in expression. The Gene Ontology (GO) enrichment analysis of DEGs in paeonol-treated *C. albicans* SC5314 biofilms as compared with untreated control showed that these genes negatively regulated by paeonol were mainly enriched in molecular function related to drug binding, transporter activity, and transmembrane transporter activity, and cell components related to the membrane, membrane part, intrinsic component of the membrane, and integral component of the membrane, as well as biological processes associated with transmembrane transport, localization, and establishment of localization (**Figure 10A**). Interestingly, among downregulated DEGs, fifteen were found to be implicated in the regulation of cell wall biogenesis (e.g., Crz2 (a zinc finger transcription factor of the Cys2His2 family), Pga62 (GPI-anchored protein 62), Kre1 (Killer toxin-resistance protein 1), biofilm formation [e.g., Crz2, Mrv8 (orf19.3908)], drug resistance [e.g., Cdr1 (pleiotropic ABC efflux transporter), Fcr1 (fluconazole resistance protein 1), Mdr1 (multidrug resistance protein 1)], and filamentous or hyphal growth [e.g., Gdh3 (glutamate dehydrogenase), Sok1 (orf19.451), Sfl1 (transcription factor), Rme1 (zinc finger protein), Ras2 (Ras-like protein 2), Kip1 (kinesin-like protein), Pth2 (peptidyl-tRNA hydrolase 2), Tpk1 (orf19.4892)] (**Figure 10C**). In addition, increased gene expression was involved in glucose transporter (e.g., Hgt17, glucose transporter), the transport of lactate (e.g., Jen1, orf19.7447), and the uptake of glycerophosphocholine (e.g., Git4, glycerophosphocholine permease) (**Figure 10C**).

For paeonol-treated *C. neoformans* H99 biofilms, a total of 485 differentially expressed genes were found, among which 236 genes displayed a decrease in expression and 249 demonstrated an increase in expression. Contrary to the findings of paeonol-treated *C. albicans* SC5314 biofilms, the GO enrichment analysis of DEGs in paeonol-treated *C. neoformans* H99 biofilms compared with the untreated control revealed that these genes positively regulated by paeonol were mainly enriched in molecular functions related to transporter activity, transmembrane transporter activity, and oxioreductase activity, and cell components related to the membrane, membrane part, intrinsic component of the membrane, integral component of the membrane, as well as biological processes related to transmembrane transport, localization, establishment of localization, and oxidation-reduction process (**Figure 10B**). Interestingly, eleven overexpressed genes were related to the lipid metabolic process. Among them, the expression levels of eight genes involved in the ergosterol (Erg) biosynthetic pathway encoding for Erg1 (squalene monooxygenase), Erg2 (C-8 sterol isomerase), Erg3 (C-5 sterol desaturase), Erg5 (C-22 sterol desaturase), Erg7 (lanosterol synthase), Erg11 (lanosterol 14 alpha-demethylase), Erg25 (C-4 methylsterol oxidase), and Sre1 (a regulator of sterol homeostasis) were upregulated. Moreover, Rds2 (a regulator of drug sensitivity) gene expression levels related to capsule production were upregulated, whereas Pdr802 (a putative Zn2-Cys6 zinc-finger transcription factor) was significantly downregulated after exposure to paeonol (**Figure 10D**). In addition, to verify the changes in gene expression from the RNA-sequencing data, several DEGs regulated by paeonol were examined using qRT-PCR. As shown in **Figures 10E, F**, the qRT-PCR results of the DEGs tested after paeonol treatment were consistent with RNA-sequencing data.

## Discussion

Fungi, including *C. albicans* and *C. neoformans*, are ubiquitous microorganisms with worldwide distribution. Increasing data have demonstrated that *C. albicans* and *C. neoformans* possess various abilities to adhere to and invade different types of host cells (Mayer et al., 2013; Sheppard and Filler, 2014). During infection, *C. albicans* and *C. neoformans* form a complex community architecture known as a biofilm, which allows them to spread through tissues and reshape their phenotypic and functional characteristics to protect themselves from immune attacks (Kernien et al., 2017). With the rise of antifungal resistance, treating *C. albicans* and/or *C. neoformans*-associated infections caused by biofilms with conventional antifungals is no longer as effective, thereby leading to enhanced mortality or severe toxicity (Martinez and Casadevall, 2006; Delattin et al., 2014; Nobile and Johnson, 2015). Further compounding this issue is the very few new antifungal agents available to fight deadly fungal infections in the pipeline. The current situation highlights the need to search for novel, alternative, and natural-based antifungal agents for fungal infections. Recently, plant-derived natural products used in traditional Chinese medicine have attracted much attention from researchers because they tend to show less resistance and are widely use in clinical practice (Zhao et al., 2019).

The data obtained in the present study exhibited potent antifungal and antibiofilm activities of paeonol against mono- and dual-species cultures of *C. albicans* SC5314 and *C. neoformans* H99. The MIC values of paeonol for planktonic cells of mono- and dual-species *C. albicans* SC5314 and *C. neoformans* H99 cultures were 250, 250, and 500 μg ml^−1^, respectively. Recently, *in vitro* evaluation of antifungal activity experiments identified several different types of natural products derived from traditional Chinese herbs against *C. albicans* and *C. neoformans*, including magnolol, plagiochin E, baicalin, curcumin, silibinin, *Amyris balsamifera* essential oil, aloe emodin, barbaloin, etc., with MIC values ranging from 2 to 1,024 μg ml^−1^ (Agarwal et al., 2000; Bang et al., 2000; Martins et al., 2009; Wang et al., 2015; Kumari et al., 2017; Yun and Lee, 2017; Brilhante et al., 2020). This discrepancy in MIC values may be attributed to the mechanisms of action of natural substances.

In recent years, a considerable number of natural molecules have been exploited in medical fields as potential antifungals and have been found to possess therapeutic effects against opportunistic fungal pathogens such as *C. albicans* and *C. neoformans* (Zida et al., 2017; Aldholmi et al., 2019). These natural compounds belong to different types of secondary plant metabolites, including essential oils, flavonoids, alkaloids, terpenoids, phenols, etc. Their antifungal mechanisms of action may be involved in antivirulence and antibiofilm properties, inhibiting cell wall biosynthesis or disrupting cell membrane, and inducing cell apoptosis (Zida et al., 2017; Iyer et al., 2021). Fungi cell membranes are predominantly composed of sterols, glycerophospholipids, and sphingolipids, and they are a multifaceted membrane that serves as the material basis for various functional proteins while also maintaining cell integrity (Iyer et al., 2021). Therefore, some antifungal agents usually target the cell membrane. In this study, to reveal the potential antifungal mechanism of action of paeonol, the visualization of cell membrane integrity of paeonol-treated *C. albicans* SC5314 and *C. neoformans* H99 was performed using CLSM by coupling with different staining protocols. In this study, CLSM images suggested that paeonol at a MIC may damage the cell membrane integrity of *C. albicans* SC5314 and *C. neoformans* H99 single and mixed-species, as evidenced by a similar increase in bright red and blue fluorescences in a concentration-dependent manner, revealing increased membrane permeability in response to an increase in paeonol exposure. Similar studies have demonstrated that a lot of natural products present robust fungistatic effects by damaging the integrity of the cell membrane. For instance, (−)-olivil-9′-O-β-d-glucopyranoside from *Sambucus williamsii* exerts antifungal activity against *C. albicans* by disrupting the cell membrane, as evidenced by the increased influx of stains (Lee et al., 2014). Robbins et al. (2016) also demonstrated that ibomycin possesses antifungal activity by impeding cell membrane function.

Live-cell imaging is widely performed by analyzing the influx of fluorescent molecules using a fluorescence microscope, which can provide vital information about cellular integrity. In this study, the influx of gatifloxacin with intrinsic fluorescence was used to investigate cell integrity using CLSM. Our findings further suggested that paeonol promoted gatifloxacin to gain more access to the cell interior due to the increased cellular permeability. A similar mode of analysis was reported previously in astacidin 1-treated *C. albicans*, in which fluorescein isothiocyanate-labeled dextrans (FDs) of various sizes were added to *C. albicans* cells exposed to astacidin 1 to evaluate the average size of pores formed in the cell membrane by measuring the influx of fluorescent molecules (Choi and Lee, 2014). The results showed that astacidin 1 caused the influx of FD4, indicating that astacidin 1 may exert antifungal activity *via* pore-forming action on the fungal membrane (Choi and Lee, 2014). Interestingly, the use of gatifloxacin with intrinsic fluorescence characteristics may be more beneficial and simple than a synthesis process of small‐molecule fluorescent probes.

FESEM was employed to assess the changes in cell morphology of *C. albicans* SC5314 and *C. neoformans* H99 in response to paeonol. The findings of the FESEM study confirmed that cells exposed to paeonol showed a significant increase in the number of cells with deformed and damaged cell surfaces. These surface structural disorders of cells may be attributed to a decrease in the content of cell wall constituents in the presence of paeonol. Similarly, Li et al. claimed that paeonol disrupts the integrity of *Aspergillus flavus* cell walls *via* inhibiting the biosynthesis of β-1,3-glucan (Li et al., 2021). Moreover, the microplate-based assay was employed to determine cell viability by evaluating the intracellular conversion of the green fluorescent FUN^®^ 1 dye to yellow or red fluorescent intravacuolar structures, and only viable and metabolically active cells possess the ability to perform this conversion. In this study, similar results induced by paeonol were also observed with FUN^®^ 1 and CWS analysis for mono- and dual-species cultures of *C. albicans* SC5314 and *C. neoformans* H99, in which the numbers of metabolically inactive or dead fungal cells were increased with increasing paeonol concentrations in a concentration-dependent manner. The viability assay results provided strong evidence that paeonol indeed exerts fungicidal effects on the mono- and dual-species cultures of *C. albicans* SC5314 and *C. neoformans* H99 *via* damaging the membrane integrity of the fungal cell.

Next, the antibiofilm efficacy of paeonol and its mechanism of action against *C. albicans* SC5314 and *C. neoformans* H99 mono- and dual-species were assessed using FESEM, CLSM, RNA-sequencing, etc. Biofilm formation is a multistep process that involves initial cell attachment to a surface, the formation of a microcolony, and finally ending with maturation followed by detachment. Therefore, inhibiting initial cell adhesion on surfaces and inducing biofilm dispersal could be effective strategies for combating biofilm-associated infections (Fuentefria et al., 2017). The present results showed that subinhibitory concentrations of paeonol effectively impede fungal cell attachment to the glass surface by partly reducing the metabolic activities of *C. albicans* SC5314 and *C. neoformans* H99 mono- and dual-species, and thus reducing the amount of biofilm formed by mono- and dual-species of *C. albicans* SC5314 and *C. neoformans* H99. Similarly, the essential oil extracted from *Rosmarinus offcinalis* demonstrated potent anti-adherent potency on biofilm by *C. albicans* through significant cell disruption (Cavalcanti et al., 2011). Moreover, one of the typical properties of cells with mature biofilms is that much higher concentrations of antimicrobial agents are required to inactivate biofilm-related cells compared with planktonic cells. Our results further demonstrated that, in addition to actively dispersing mature biofilms of *C. albicans* SC5314 and *C. neoformans* H99, higher concentrations of paeonol can damage cells within mature biofilms. Together, these observations indicated that paeonol could cause the dispersal of mature biofilms by *C. albicans* SC5314 and *C. neoformans* H99 mono- and dual-species into planktonic states and by enabling antifungal agents that work on planktonic fungal cells to take effect. In addition, paeonol inhibited yeast-hyphae morphological transformation of *C. albicans* SC5314, and impaired capsule and melanin production of *C. neoformans* H99.

RNA-sequencing and qRT-PCR further revealed that paeonol interfered with the biofilm formation and formation of hyphae of *C. albicans* SC5314 through primarily downregulating the expression of biofilm biosynthesis and hypha-specific genes, including Crz2, Pga37, Mrv8, Gdh3, Sok1, Sfl1, Rme1, Ras2, Kip1, Pth2, and Tpk1. Crz2 is part of a set of adherence regulators required for the adhesion of *C. albicans* to abiotic substrates, some of which modulate the expression of cell-surface targets of adherence regulators and hyphal growth or virulence genes (Finkel et al., 2012; Znaidi et al., 2018). Previous studies showed that overexpression of Pga37 caused enhanced occupancy of the multistrain biofilm (Cabral et al., 2014), and Mrv8 encodes a four-pass transmembrane protein unique to the closely related pathogens *C. albicans* and *Candida dubliniensis*, for which Mrv8 is dispensable for the initial filamentation, adhesion, mycelial maturation, and invasion events associated with *C. albicans*-epithelial infection (Costa et al., 2020). Similarly, overexpression of Sok1 overcomes the farnesol-mediated inhibition of hyphal initiation (Lu et al., 2014), and mutants lacking both Tpk1 alleles show defective hyphal morphogenesis on solid inducing media (Bockmuhl et al., 2001). In addition, deletion of Ras2 reduces filamentous growth of *C. albicans* (Feng et al., 1999). Riccardin D extracted from the liverwort *Dumortiera hirsute* affected the biofilm formation and hyphae formation of *C. albicans* by downregulating the expression of biofilm formation and hypha-specific genes (Li et al., 2012). Costa et al. (2020) reported that FESEM images revealed that the mature biofilm of *C. albicans* coating the substrate surface contains yeast, pseudohyphae, and hyphal cells (Lattif et al., 2011). Therefore, we assume that paeonol affected the biofilm formation of *C. albicans* by presumably downregulating the expression levels of initial filamentation, adhesion, and growth-related genes.

In the paeonol-treated *C. neoformans* H99 context, RNA-sequencing and qRT-PCR revealed that when *C. neoformans* H99 biofilms were treated with paeonol, the expression of genes involved in ergosterol biosynthesis was upregulated significantly, indicating drastic variation in the sterol profile upon paeonol treatment. Previous studies showed that the variation in the sterol profile of biofilm cells affects the ability of *C. albicans* to form biofilms (Mukherjee et al., 2003). For example, Mukherjee et al. (2003) performed gas–liquid chromatography to obtain an insight into the levels of sterols between planktonic and biofilm cells and found that both biofilm and planktonic *C. albicans* show similar ergosterol levels at 6 h, followed by a reduction in ergosterol levels in the intermediate and mature phase of biofilms, indicating that the decrease in ergosterol as the biofilm matures is larger. Similarly, Mukherjee et al. (2003) found that the cell membranes of biofilm cells consist of a markedly lower concentration of ergosterol, especially during the later phases of biofilm growth, suggesting that the mature biofilms rely less on ergosterol for maintaining membrane fluidity (Lattif et al., 2011). Moreover, the biosynthesis of ergosterol, the major sterol in fungal cell membranes, is intricately regulated by approximately 25 known enzymes related to the ergosterol production pathway. The impact of ergosterol gene overexpression on fungal growth was remarkable. In this scenario of great complexity, Bhattacharya et al. (2018) reported that overexpression of Erg9, Erg1, Erg25, Erg27, Erg28, Erg6, and Erg2 in *Saccharomyces cerevisiae* resulted in reduced growth rates in rich medium. Based on these findings, it may be deduced that paeonol increased the levels of ergosterol of *C. neoformans* H99 by primarily upregulating the expression of genes related to the ergosterol biosynthesis pathway, probably leading to the inhibition of biofilm formation. In addition, the deletion of Hlh1 greatly enhanced melanin production in *Cryptococcus neoforman*s (Jung et al., 2015), and direct targets of Pdr802 include the quorum sensing proteins Pqp1, Opt1, and Liv3, which regulate cryptococcal brain infectivity and capsule thickness (Reuwsaat et al., 2021).

Recently, a few studies have demonstrated that *C. elegans* has been explored as an attractive model host for virulence assays of microbial pathogens, such as *C. albicans*, *C. neoformans*, *Salmonella enterica*, *Pseudomonas aeruginosa*, *Staphylococcus aureus*, and *Enterococcus faecalis* (Mylonakis et al., 2002; Marsh and May, 2012; Park et al., 2017; Elkabti et al., 2018; Kumar et al., 2020). The *C. elegans* model does not raise the same special ethical concerns as the use of vertebrate models when compared with vertebrates (Ewbank and Zugasti, 2011). In addition, due to several excellent characteristics of *C. elegans*, such as a rapid generation time and its transparency, rendering the use of fluorescent reporter genes *in vivo* straightforward, *C. elegans* has been exploited to assess the compound’s antifungal activity and the compound’s cellular toxicity (Kim et al., 2020). In this study, *C. elegans* killing assays were conducted to examine the effectiveness of paeonol as a potential antifungal chemical compound, and paeonol effectively prolonged *C. elegans’s* survival when exposed to *C. albicans* SC5314 or *C. neoformans* H99 using a liquid killing assay. These data indicated that paeonol had beneficial effects on *C. elegans*’ lifespan and significantly prolonged survival in *C. albicans* SC5314 or *C. neoformans* H99-infected worms, suggesting that the *in vivo* efficacy of paeonol may be easily estimated by fungal burden or mortality rate in infected and treated *C. elegans*.

## Conclusions

The results of the present study confirm that paeonol could be a promising antifungal agent for inactivating planktonic and biofilm cells of mono- and dual-species *C. albicans* SC5314 and *C. neoformans* H99, inhibiting biofilm formation, and dispersing mature biofilms. The transcriptome analysis elucidated some of the molecular mechanisms of paeonol treatment against *C. albicans* SC5314 and *C. neoformans* H99 biofilms. Taken together, our results suggest that paeonol shows promise in combating opportunistic fungal infections caused by *C. albicans* and *C. neoformans* mono- and dual-species biofilms.

## Data Availability Statement

The raw data supporting the conclusions of this article will be made available by the authors, without undue reservation.

## Author Contributions

WQ: conceptualization and writing—review and editing. XL: software, investigation, validation, and data acquisition. QL: software. JL and QZ: analysis and interpretation. TW: methodology and supervision. All authors listed have made a substantial, direct, and intellectual contribution to the work and approved it for publication.

## Funding

This study was supported by the Science and Technology Plan Project of Xi’an Science and Technology Bureau (2020KJRC0007), the Xi’an Weiyang District Science and Technology Project (201926), the China Postdoctoral Science Foundation (2019M653938), and the Natural Science Pre-research Fund of Shaanxi University of Science and Technology (2017BJ-48).

## Conflict of Interest

The authors declare that the research was conducted in the absence of any commercial or financial relationships that could be construed as a potential conflict of interest.

## Publisher’s Note

All claims expressed in this article are solely those of the authors and do not necessarily represent those of their affiliated organizations, or those of the publisher, the editors and the reviewers. Any product that may be evaluated in this article, or claim that may be made by its manufacturer, is not guaranteed or endorsed by the publisher.
