# Supplementary material for: Antifungal and Antibiofilm Efficacy of Paeonol Treatment Against Biofilms Comprising Candida albicans and/or Cryptococcus neoformans
Source: Front Cell Infect Microbiol. 2022 May 20;12:884793. doi: 10.3389/fcimb.2022.884793 (PMC9163411; doi:10.3389/fcimb.2022.884793)
Supplement: Supplementary file 1 [file Table_1.doc]

Table S1 Primers for *C. albicans* (CA) and *C. neoformans* (CN) genes used in real-time PCR assay.

| Primer | Gene product | Sequence (5’-3’) | References |
| --- | --- | --- | --- |
| CN-Act F | Actin | TTGTCCTCCCCAATCTTCAC | Derengowskia., et al. 2013, 12(5): 761-774. |
| CN-Act R |  | CGGCGACTTCTTCTTCCATA |  |
| CN-Erg3 F | ERG3 | CCCAGTCCCTTCCTTACCAT |  |
| CN-Erg3 R |  | TCATATCACCGTCGTGGATG |  |
| CN-Erg11F | ERG11 | TCCCATCCACTCGATCTACC |  |
| CN-Erg11 R |  | CCCTTGGGGATGATGTATTG |  |
| CN-PDR802 F | PDR802 | GTCGGCAGTTCTCATCATC | Reuwsaat., et al. 2021, 12(2):e03457-20. |
| CN-PDR802 R |  | TCATCCTCAGCATTCTTGG |  |
| CN-Sre1 F | SRE1 | | ATGTTCCTGCTTCCAGAGCC | | --- | | This study |
| CN-Sre1 R |  | GCTGCATTGGTTGTTGCTGA |  |
| CN-Rds2 F | RDS2 | GCAAGAACCTCAGCAGCAAC |  |
| CN-Rds2 R |  | CTGGGGACGAAGTTGGAGTC |  |
| CA-Act1 F | Actin | CAAACCACTTTCAACTCCATCA | Cheng., et al. 2021, 24(2): 233–242. |
| CA-Act1 R |  | GAACCACCAATCCAGACAGAG |  |
| CA-Cdr1 F | CDR1 | ATCCAACACCAGGGAAACTT | Khosravi., et al. 2016, 2(4):24-29. |
| CA-Cdr1 R |  | TCGCAACACCATACCTCACT |  |
| CA-Mdr1 F | MDR1 | TACGCGGGTTCTTTGTTGTAT |  |
| CA-Mdr1 R |  | GATAATGTTTAGCAAGCCGAGGA |  |
| CA-Gdh3 F | GDH3 | GGGTGGTGGTAAAGGTGGTT | This study |
| CA-Gdh3 R |  | ATCACCAGCTGGAACATCGG |  |
| CA-Crz2 F | CRZ2 | CAGCCACGTCATCCTCCATT |  |
| CA-Crz2 R |  | CATGCAGTCGAGCAAATCGT |  |
| CA-Gca2 F | GCA2 | GCGTGCTTTCAACTGGCAAT |  |
| CA-Gca2 R |  | \| AGGAGCAGCCAAAGTTTCGT \| \| --- \| |  |

**References**:

Lorena da S. Derengowskia, Hugo Costa Paes, Patrícia Albuquerquea, Aldo Henrique F. P. Tavaresa, Larissa Fernandesa, Ildinete Silva-Pereira and Arturo Casadevall. (2013) The Transcriptional Response of *Cryptococcus neoformans* to Ingestion by *Acanthamoeba castellanii* and Macrophages Provides Insights into the Evolutionary Adaptation to the Mammalian Host. *Eukaryotic Cell*, 12(5): 761-774. [doi: 10.1128/EC.00073-13](https://doi.org/10.1128/EC.00073-13)

Julia C.V. Reuwsaat, Daniel P. Agustinho, Heryk Motta, Andrew L. Chang, Holly Brown, Michael R. Brent, Livia Kmetzsch, Tamara L. Doering. (2021) The Transcription Factor Pdr802 Regulates Titan Cell Formation and Pathogenicity of *Cryptococcus neoformans*. *mBio*, 12(2):e03457-20. [doi:](https://doi.:/) 10.1128/mBio.03457-20.

Rong Cheng, Qiang Xu, Fangfang Hu, Hongling Li, Bin Yang, Zonggang Duan, Kai Zhang, Jianwei Wu, Wei Li, Zhenhua Luo. (2021) Antifungal activity of MAF-1A peptide against *Candida albicans*. *Int Microbiol*, 24(2): 233–242. doi: 10.1007/s10123-021-00159-z

K Khosravi Rad, M Falahati, M Roudbary, S Farahyar, S Nami. (2016) Overexpression of MDR-1 and CDR-2 genes in fluconazole resistance of *Candida albicans* isolated from patients with vulvovaginal candidiasis. *Curr Med Mycol*, 2(4):24-29. doi: 10.18869/acadpub.cmm. 2.4.24.
